# Tentaclins—A Novel Family of Phage Receptor-Binding Proteins That Can Be Hypermutated by DGR Systems

**DOI:** 10.3390/ijms242417324

**Published:** 2023-12-10

**Authors:** Ivan K. Baykov, Artem Y. Tikunov, Igor V. Babkin, Valeria A. Fedorets, Elena V. Zhirakovskaia, Nina V. Tikunova

**Affiliations:** Federal State Public Scientific Institution «Institute of Chemical Biology and Fundamental Medicine», Siberian Branch of the Russian Academy of Sciences, 630090 Novosibirsk, Russia

**Keywords:** bacteriophage, genome sequence, diversity-generating retroelement, C-type lectin, Ig-like domain, receptor-binding protein, tentaclin, adhesin

## Abstract

Diversity-generating retroelements (DGRs) are prokaryotic systems providing rapid modification and adaptation of target proteins. In phages, the main targets of DGRs are receptor-binding proteins that are usually parts of tail structures and the variability of such host-recognizing structures enables phage adaptation to changes on the bacterial host surface. Sometimes, more than one target gene containing a hypermutated variable repeat (VR) can be found in phage DGRs. The role of mutagenesis of two functionally different genes is unclear. In this study, several phage genomes that contain DGRs with two target genes were found in the gut virome of healthy volunteers. Bioinformatics analysis of these genes indicated that they encode proteins with different topology; however, both proteins contain the C-type lectin (C-lec) domain with a hypermutated beta-hairpin on its surface. One of the target proteins belongs to a new family of proteins with a specific topology: N-terminal C-lec domain followed by one or more immunoglobulin domains. Proteins from the new family were named tentaclins after TENTACLe + proteIN. The genes encoding such proteins were found in the genomes of prophages and phages from the gut metagenomes. We hypothesized that tentaclins are involved in binding either to bacterial receptors or intestinal/immune cells.

## 1. Introduction

A diversity-generating retroelement (DGR) is a prokaryotic molecular system that provides hypermutation in a certain variable region of the target gene, which is a part of the DGR cassette [1]. Both bacteria and phages use this mechanism for rapid adaptation to permanent changes in the environment [2,3]. Probably, bacteria can also use DGR cassettes to increase the diversity of proteins that perform protective or immune functions [4]. Phages mainly use this mechanism to modify their receptor-binding proteins for maintaining the ability to infect host bacteria when certain components change on the cell surface [5,6,7,8,9]. In particular, this is observed for phages infecting bacteria that inhabit the intestine [10,11,12,13]. The habitat of such bacteria often changes depending on nutrition, the health of the macroorganism, and some external factors that can lead to modification of the state of bacteria including their surface molecules.

The signature components of the DGR cassette are the reverse transcriptase (RT) gene, template repeat (TR) with a length of 100–150 bp, and target gene containing a variable locus similar to the TR that is called a variable repeat (VR). At the 3′-end of the VR, the initiating of the mutagenic homing (IMH) sequence is located, whereas the IMH* sequence that is not identical to the IMH is at the 3′-end of TR [1,2]. In addition, the DGR cassette usually contains the accessory gene that encodes the accessory variability determinant (Avd) protein or its analog, which is essential for the DGR activity [6,14]. The molecular mechanism of DGR machinery is not completely clear. It has been established that the key stage is the reverse transcription of the RNA copy of TR by the RT, which substitutes only the adenine nucleotides during the process of hypermutagenesis [1,7]. Then, the mutated TR copy replaces VR in the target gene in the process of retrohoming. In the DGR cassette of the *Bordetella* phage BPP-1 that is the first described DGR, the target gene *mtd* encodes the major tropism-determinant protein. This protein is connected to the distal end of tail fibers and is responsible for the recognition of receptor structures on the surface of the host cell [5,6,7,15]. So, modification of some receptor-binding proteins facilitates the adaptation of phage structures to possible changes in the surface structures of the cell [7,16].

It has been shown that approximately 13% of the analyzed prokaryotic DGR cassettes contain two target genes [1] and up to eight similar target genes have been found in DGRs of *Treponema denticola* and *Stenotrophomonas* sp. [17,18]. One hypothesis suggests that such genes occurred as a result of duplication and serve to adapt the organism to changing conditions due to the parallel specialization of each of the gene variants [18,19]. Limited data on phases containing more than one target gene are known [1].

In this study, we found that several phage genomes that were assembled from human gut metagenomes maintain DGR cassettes with two different target genes. One of the target genes contained VR in the 5′-part of the gene, whereas in most DGR cassettes VR is located at the 3′-end of the target gene. Bioinformatic analysis indicated that both target proteins have different topology and the protein with VR located in its N-terminal part is a member of a new large family of proteins that were named tentaclins. Notably, hundreds of genes encoding tentaclins were found in the human gut microbiomes and bacterial genomes.

## 2. Results

### 2.1. Search for Phage DGRs with Two Target Genes

**Figure 1:**
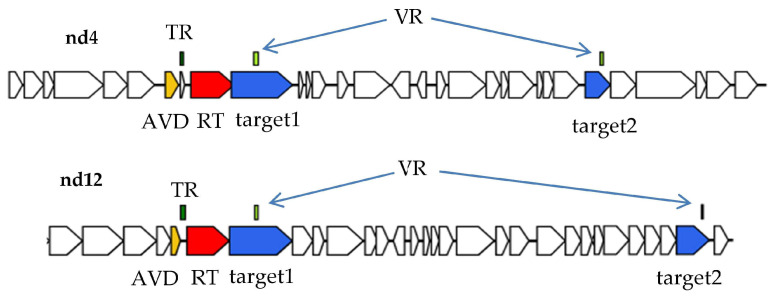
DGR cassettes from the nd4 and nd12 genomes generated using myDGR service. Target genes are colored in blue, reverse transcriptase (RT) genes are red, accessory protein AVD genes are orange. TR—template repeat, VR—variable repeat.

In order to find phage DGR cassettes with two target genes, several gut viromes of healthy people were sequenced and all assembled contigs were screened for the presence of essential phage genes. Contigs that contained the genes encoding both phage large terminase subunit and portal protein were selected. Then, sequences containing DGR cassettes were identified among the selected phage and prophage genomes using myDGR service [20]. Finally, complete DGR cassettes containing two target genes (target1 and target2) were found in two phage genomes named nd4 and nd12 (Figure 1). In both DGR cassettes from the nd4 and nd12 genomes, the target1 gene contained VR close to the 5′-end of the gene, whereas target2 had VR at its 3′-end like most of the known phage target genes [1].

**Figure 2:**
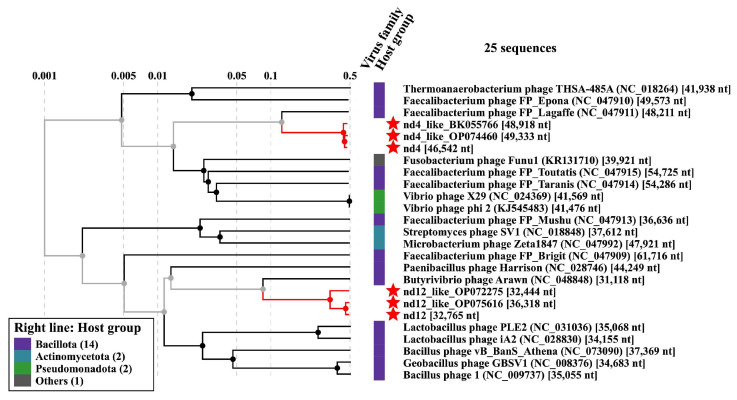
ViPTree-generated “proteomic” dendrogram of viral genome sequences based on genome-wide sequence similarities computed by tBLASTx, indicating the position of the nd4, nd12, and several nd4-like and nd12-like genomes (marked with red asterisks).

Phage genomic sequences nd4 and nd12 (46,542 bp and 32,765 bp, respectively) contained the gene encoding the tail sheath protein (Supplementary Data Tables S1 and S2) that is a signature protein of phages with myovirus morphology. Notably, nd4 and nd12 were quite distant; their nucleotide identity (NI) was calculated as 31.2%. A search for sequences related to the nd4 and nd12 phage genomes in the GenBank database revealed similar phage sequences from the human gut viromes for both studied phages (Figure 2). The size of the genomes similar to nd4 did not exceed 51 kbp (GenBank OP074837.1; query coverage 93%; NI 99%) and varied from 48.2 kbp to 49.3 kbp for the closest relatives (GenBank OP074460.1, BK055766.1, and OP074962.1; query coverage 96–97%; NI 98–99%). Therefore, the sequence of the nd4 genome can be considered almost complete. Similarly, the size of the genome that was most similar to nd12 was 36,318 bp (GenBank OP075616.1; query coverage 94%, NI 98.7%); so, the nd12 genome contained ~90% of the complete sequence. Given the small size of the nd4 and nd12 genomes, the phages can be attributed to small myoviruses. Notably, only a single nd4-like prophage genome was found among the bacterial genomes. This prophage was found in the genome of the *Flavonifractor plautii* strain VE303-08 (query coverage 84%, NI 85%). No prophages similar to nd12 were found.

### 2.2. Proteins Encoded by the Target1 Genes from the nd4 and nd12 Genomes

**Figure 3:**
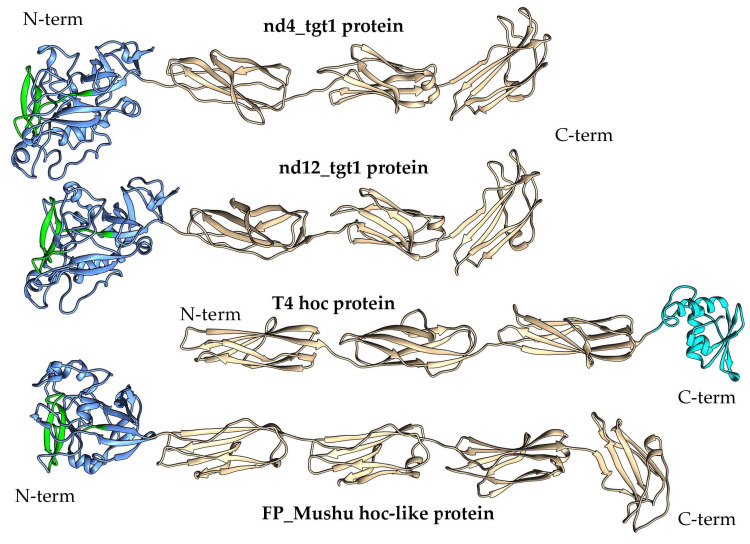
Ribbon representation of predicted 3D structures of the nd4_tgt1, nd12_tgt1, T4 Hoc proteins, and FP_Mushu phage Hoc-like protein. C-type lectin domains are in blue, Ig domains are in tan, and anchoring domain of the T4 Hoc protein is cyan. VR-encoded regions are marked with green. Three-dimensional models were predicted using AlphaFold2 and rendered using UCSF Chimera. Also see Figure S1 and Figure 6.

**Figure 4:**
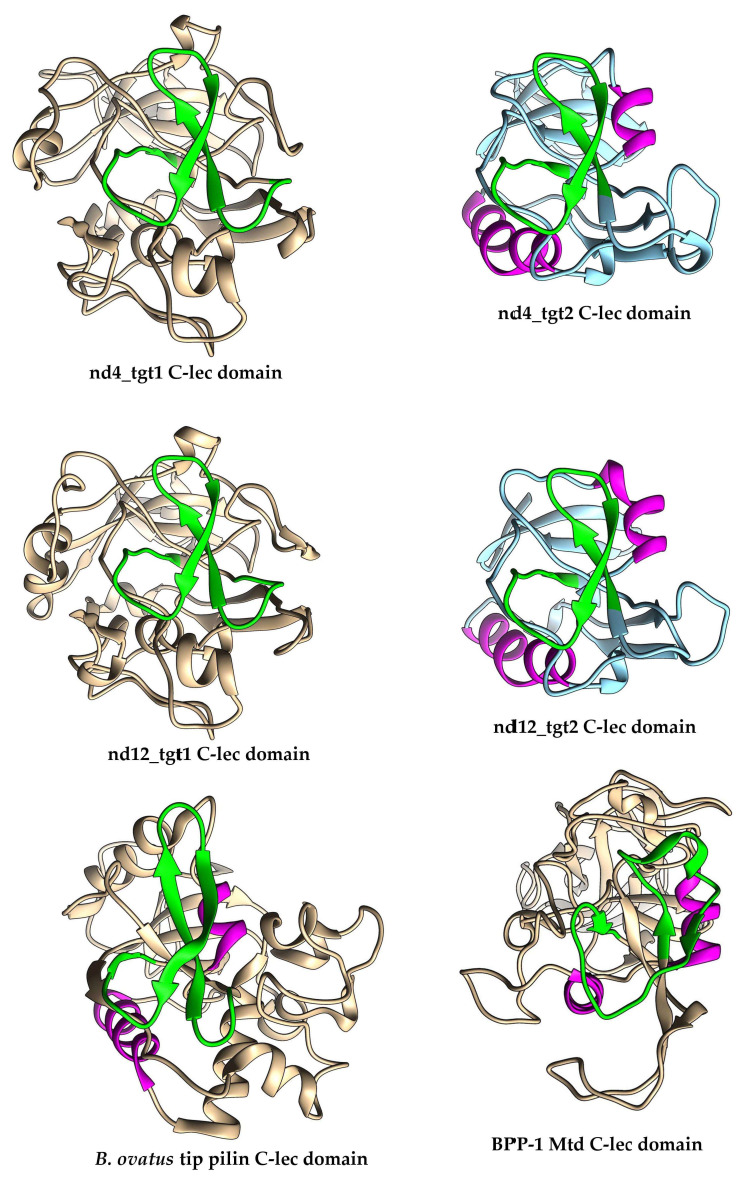
Ribbon representation of predicted 3D structures of C-lec domains of the nd4_tgt1, nd4_tgt2, nd12_tgt1, and nd12_tgt2 proteins. Experimental structures of C-lec domains of the BPP-1 Mtd protein (pdb 1YU0) and tip pilin of *Bacteroides ovatus* (pdb 4EPS) are shown for comparison. VR-encoded regions are marked with green. Highlighted alpha-helices indicate similar orientation of the molecules. Three-dimensional models were predicted using AlphaFold2, structure relaxation performed using GROMACS, and final models were rendered using UCSF Chimera.

Target genes containing VR close to the 5′-end of the gene are rare among phage DGR systems [1]. Analysis of the proteins encoded by the nd4 and nd12 target1 genes (569 and 579 aa, respectively) using AlignX (Vector NTI suite 8.0) indicated that the identity of these proteins is 47%. NCBI Conserved Domain (CD) search tool revealed that the nd4_tgt1 and nd12_tgt1 proteins contain the N-terminal DUF6273 domain of unknown function followed by the fibronectin type 3 family (Fn3) domain belonging to the Ig-like domain superfamily. Analysis using HHpred did not provide additional information. To clarify the possible function of these proteins, AlphaFold2 was applied to obtain putative three-dimensional (3D) structures of the proteins with high confidence (high pLDDT score) (Figure 3 and Figure S1). According to the models, the nd4_tgt1 and nd12_tgt1 proteins have similar 3D structures: they contain the N-terminal globular domain followed by three beta-sandwich domains that belong to the immunoglobulin (Ig) superfamily. A search for similar structures among experimentally determined structures using DALI indicated that the N-terminal DUF6273-like domain resembles the C-type lectin (C-lec) domain. Notably, VR forms a characteristic beta-hairpin type structure flanked by two additional loops located on the surface of this domain in both nd4_tgt1 and nd12_tgt1 (Figure 3 and Figure 4). Molecular dynamics relaxation for 50 ns did not reveal any significant deviations in the conformation of the hairpin during the simulation.

The presence of Ig-like domains indicated a possible structural similarity of the nd4_tgt1 and nd12_tgt1 proteins with the capsid-embedded Hoc-protein of the phage T4 (Figure 3) and some phage proteins containing Ig-like domains [3,21]. Moreover, similar proteins have been also mentioned as Hoc-like targets in DGR cassettes of the prophages FP_Mushu and FP_Brigit found in *Faecalibacterium prausnitzii* [22]. However, nd4_tgt1 and nd12_tgt1 proteins have some differences compared to the T4 Hoc-protein. According to the AlphaFold model [21], the Hoc protein of the T4 phage does not contain globular N-terminal lectin domain and the structure of the C-terminal capsid-anchoring domain is also different. Modeling using AlphaFold2 showed that the Hoc-like proteins of the FP_Mushu and FP_Brigit phages have a topology more similar to that of the nd4_tgt1 and nd12_tgt1 proteins than the T4 Hoc. The Hoc-like proteins of the FP_Mushu and FP_Brigit phages also have a C-lec domain at their N-termini; however, it is followed by four Ig-like domains instead of three (Figure 3). Importantly, the aa sequence similarity of the nd4_tgt1/nd12_tgt1 proteins and the Hoc-like proteins of the FP_Mushu and FP_Brigit was low despite the similar topology. The overall protein identity for these four proteins was ~7%; however, the last ~100 aa residues forming the C-terminal Ig-like domain (Cterm_Ig domain) showed identity of ~17%. Thus, we assume that the more conserved Cterm_Ig-like domain is involved in the embedding of the tgt1 protein into the phage virion, whereas the N-terminal C-lec domain is used for binding to some receptors.

### 2.3. Comparative Analysis of the nd4_tgt1 and nd12_tgt1 Proteins

**Figure 5:**
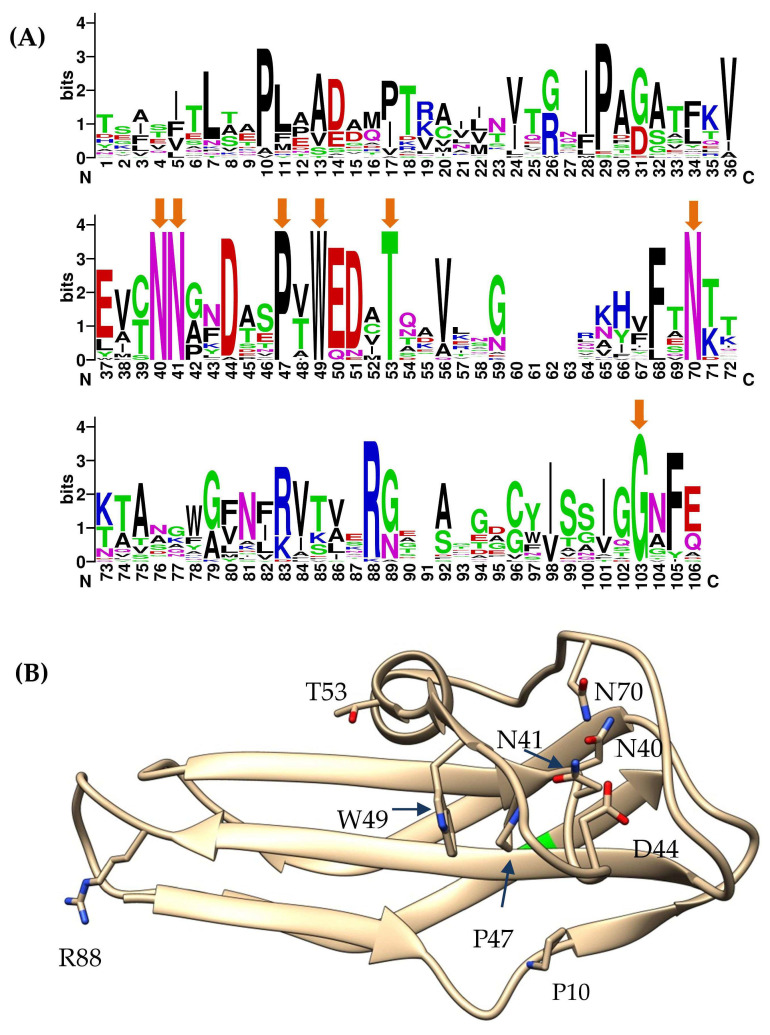
Conserved residues of the Cterm-Ig domain. (**A**) Weblogo (weblogo.berkley.edu, accessed on 1 October 2023) diagram representing consensus sequence of the Cterm-Ig domain of 29 selected proteins containing this domain. Orange arrows indicate 100% conserved aa residues. (**B**) Ribbon view of predicted nd12_tgt1 Cterm-Ig domain structure. Residues are numbered according to Weblogo diagram.

Given the high similarity of Cterm_Ig domains of the nd4_tgt1, nd12_tgt1, and the Hoc-like proteins of the FP_Mushu and FP_Brigit phages, sequences of their Cterm_Ig domains were used to find similar proteins using BLASTp search. As a result, 912 heterogeneous sequences with various degrees of similarity were extracted. Notably, a clear consensus of seven conservative aa residues was found in the Cterm-Ig regions, despite the low similarity of these regions (Figure 5A). Most of the conservative residues were located close to each other and formed a specific structure (Figure 5B).

Some of these 912 Cterm_Ig domain-containing sequences were analyzed using AlphaFold2. The obtained results indicated that even proteins with the lowest aa identity (~25%) and a low expectation value (~0.05) had a topology similar to the nd4_tgt1 and nd12_tgt1 proteins. Importantly, all analyzed proteins had the identified aa consensus in their last 100 aa sequences. Of these 912 sequences, 329 were phage sequences (up to 649 aa) encoded mainly by the metagenome-assembled genomes (MAGs); the rest sequences (up to 1445 aa) were found in bacteria (mainly in *Brevibacillus* spp. and *Bacillus badius*).

**Figure 6:**
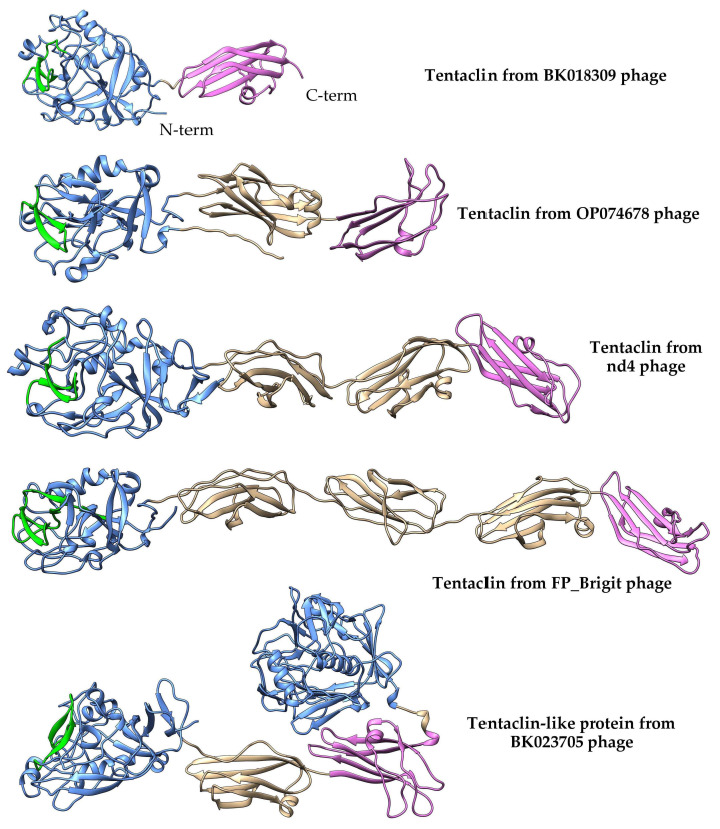
Tentaclins from various gut phage metagenome sequences. Models were generated using AlphaFold2. C-type lectin domains are in blue, beta-hairpins are in green, C-terminal anchoring Ig domains are in pink, and other Ig domains are in tan. Also see Figure S1 for pLDDT-colored models.

So, a large group of phage proteins was discovered. Since all of them contained a C-lec domain at the N-terminus, and the vast majority of C-lec domains bind polysaccharides or proteins [23], these proteins are probably receptor-binding ones. In addition, these proteins contain the Ig-like domains that are connected by unstructured regions; so, the proteins are possibly flexible like the muscle protein titin [24] or bacterial adhesins—invasin and intimin [25]. Taking into consideration the prevalence of such proteins and their possible flexibility, these proteins were named tentaclins after (TENTACLe + proteIN). Despite a certain size variability, the specific features of tentaclins are the presence of the N-terminal C-lec domain with the characteristic beta-hairpin structure and several Ig-like domains with the C-terminal domain containing a particular consensus (Figure 5 and Figure 6).

The number of Ig-like domains that occur in the tentaclins of phages and bacteria varied. All phage tentaclins contained from one to four Ig domains (Figure 6) and had a size from 313 aa to 649 aa. In addition, a phage genome (BK023705) was found that encoded a tentaclin-like protein with a size of 830 aa. This protein contained an additional C-lec domain at the C-terminus. This C-lec domain was similar (~60% identity) to one of the tail collar domains of myoviruses. The anchoring Cterm-Ig domain presumably required for tentaclin attachment to the virion was also found in this tentaclin-like protein (Figure 6). As for bacteria, their genomes encoded both phage-like tentaclins ranging from 313 aa to 650 aa and more complex tentaclin-like proteins (Figure S3). In *Brevibacillus* spp., the genes encoding tentaclin-like proteins up to 1445 aa were identified in addition to genes encoding “ordinary” tentaclins with a size of ≤650 aa.

### 2.4. Diversity of Phages Containing the Tentaclin Genes

**Figure 7:**
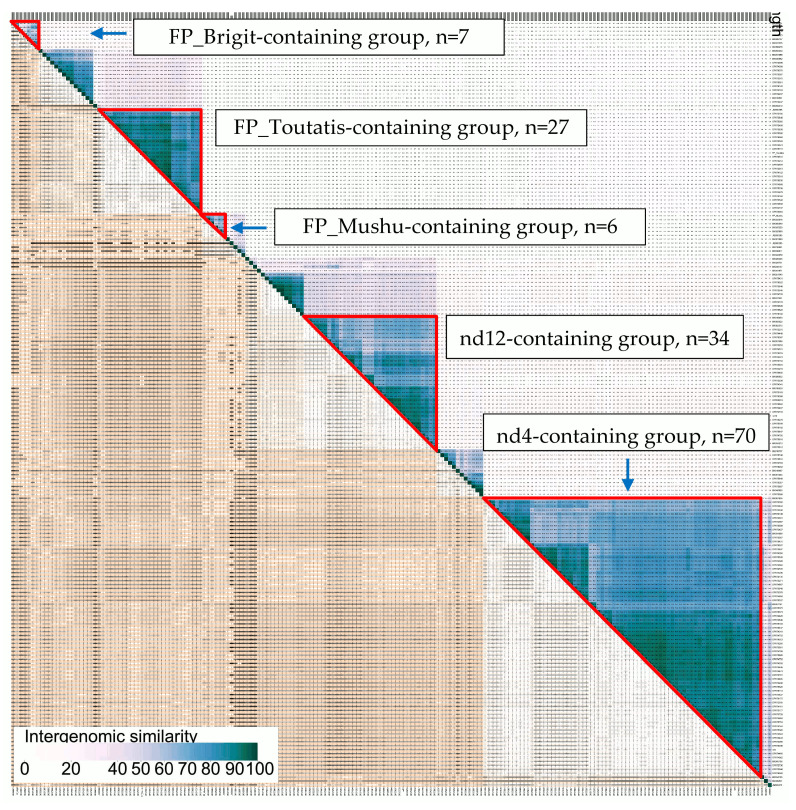
VIRIDIC heatmap indicating intergenomic similarity between phage sequences containing tentaclin genes. In this figure, 195 sequences out of 373 are shown. Also see Figure S2.

To analyze the diversity of phages containing the tentaclin genes, 373 tentaclin aa sequences annotated as phage proteins were extracted from the GenBank non-redundant protein database (nr) using BLASTp. For these sequences, the corresponding phage genomes were selected and grouped using VIRIDIC (Figure 7 and Figure S2).

The majority of these sequences were MAGs, with the exception of the previously described prophages from *Faecalibacterium prausnitzii* [22]. Grouping criteria were chosen as at least 40% intergenomic similarity (IS) with any member of the group and at least 60% IS with at least one member of the group. The nd4 phage was part of the largest group that included 70 sequences (~19% of all sequences). The nd12 phage was part of the second largest group containing 34 sequences (~9% of all sequences). The FP_Toutatis phage formed the third group of 27 sequences. The FP_Mushu and FP_Brigit phages grouped with five and six MAGs, respectively (Figure 7). The remaining sequences were unique or they formed small groups (Figure S2). Since the intergenomic similarity between the phage genomes from different groups in most cases was less than 10%, it can be concluded that the tentaclin genes occur in phages that are distant from each other and belong to different sub-families.

From five groups containing at least ten phage genomes, several sequences were randomly selected and analyzed for the presence of DGR cassette (Figure S4). It was shown that each analyzed phage genome contained the tentaclin gene as part of its DGR cassette.

### 2.5. Proteins Encoded by the Target2 Genes from the nd4 and nd12 Genomes

As for the target2 gene that contains VR at the 3′-end, we expected that this gene would encode a protein resembling the Mtd protein of the phage BPP-1. This would be in good agreement with the fact that small myoviruses FP_Lagaffe and FP_Epona found in the genomes of the *Faecalibacterium prausnitzii* strains contain *mtd*-like genes as part of their DGR cassettes [22]. However, the analysis using HHpred did not reveal a significant similarity of the secondary structure of the nd4_tgt2 and nd12_tgt2 proteins with proteins from the PBD database, including the Mtd protein. According to the NCBI CD-search, both nd4_tgt2 and nd12_tgt2 proteins contain only the DUF6273 domain, whereas the identity between the proteins was only 24%.

**Figure 8:**
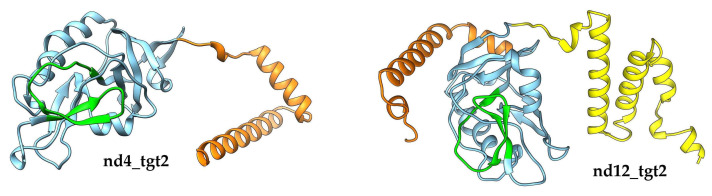
Putative 3D models of the nd4_tgt2 and nd12_tgt2 proteins. C-type lectin domains are in blue, N-terminal alpha-helices are yellow, and C-terminal alpha-helices are orange. VR-encoded regions are marked with green. Three-dimensional models were predicted using AlphaFold2 and rendered using UCSF Chimera. Also see Figure S1.

AlphaFold2 modelling indicated that nd4_tgt2 and nd12_tgt2 proteins consist of a single globular domain flanked by short alpha-helix regions (Figure 8). Analysis of the AlphaFold models of the nd4_tgt2 and nd12_tgt2 proteins using DALI indicated that the globular domains exhibited similarity with the C-type lectin domain, as was found for the N-terminal domains of the tentaclins from nd4 and nd12 (Figure 4). Notably, Mtd_BPP-1 protein that mediates binding to the bacterial receptor pertactin also contains a C-lec domain with VR at the C-terminus [16]. However, nd4_tgt2 and nd12_tgt2 proteins show substantial differences from the Mtd_BPP-1 protein: they are shorter (234 aa for nd4_tgt2 and 294 aa for nd12_tgt2 versus 381 aa for Mtd_BPP-1); they do not have a beta-sandwich domain and N-terminal beta-prism, by which the Mtd_BPP-1 trimer presumably attaches to the tail fiber protein [15,26]. Nevertheless, we suppose that the nd4_tgt2 and nd12_tgt2 proteins might perform a function similar to the Mtd_BPP-1 protein despite the differences.

The nd12_tgt2 protein contains an additional alpha-helical motif at the N-terminus, which is present in some related phages but absent in the orthologous nd4_tgt2 protein (Figure 8, yellow part). In this alpha-helical motif, InterproScan recognized a DUF3310-like motif that is found in phage and bacterial proteins. It is not yet clear whether this motif is involved in the formation of multimeric complexes or if it forms an interface for interaction with other phage proteins in the same way as the beta-prism domain of Mtd_BPP-1 interacts with the tail fiber protein.

It should be noted that VRs in the nd4_tgt2 and nd12_tgt2 proteins also form characteristic beta-hairpin structures on the surface of the C-lec domain as in the nd4 and nd12 (Figure 4). Strikingly, the shape of the hairpin structure in both target proteins (nd4 and nd12 tentaclins and nd4_tgt2/nd12_tgt2) is similar despite the different aa sequences, domain sizes, and folding details (Figure 4 and Figure S5). In all studied C-lec domains, the hairpins occupy a considerable part of the surface (Figure S6) and possibly form the receptor-binding region of these proteins. This can explain how one TR can be used as a template for hypermutagenesis of two different proteins despite the differences in their topology. Notably, VR in the Mtd_BPP-1 protein that is also subjected to hypermutagenesis has a different conformation—a loop containing a short beta strand (Figure 4). However, the structure of the C-lec domain of the terminal pilin of *Bacteroides ovatus* (pdb 4EPS) shows a beta-hairpin that is similar to that of the tentaclins and tgt2 proteins. This fact indicates that C-lec domains with the beta-hairpin motif, which were found in phage and bacterial proteins, might perform similar functions.

### 2.6. Analysis of the Hypermutagenic Potential of TRs from nd4-like and nd12-like Phages

It was analyzed whether there is a similarity between beta-hairpins sequences encoded by VR1 and VR2 in two target genes from the same phage genome. These VR sequences originated from the same TR during hypermutagenesis and the analysis of aa substitution in both VR1 and VR2 was of particular interest. If such a similarity could be detected, it would suggest that both target proteins bind to the same receptor. To test this hypothesis, TR and VR sequences from the nd4, nd12, and related genomes were involved in the analysis.

**Figure 9:**
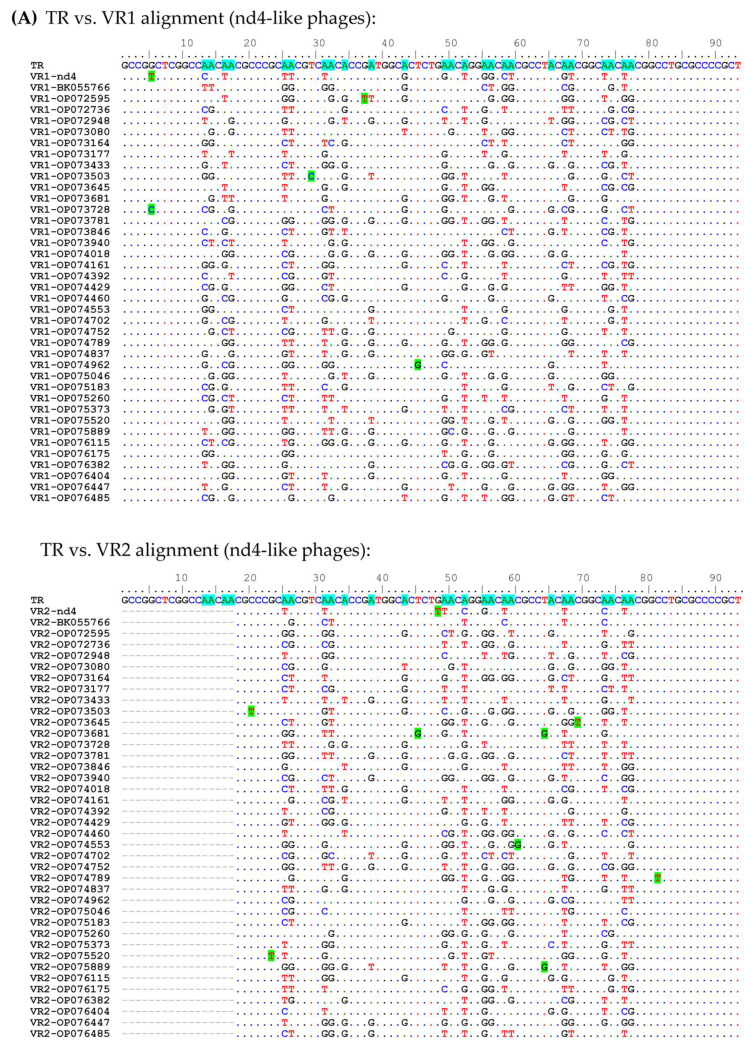

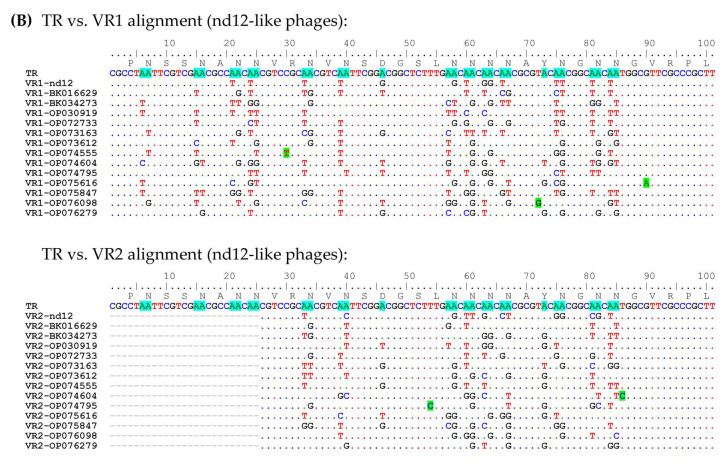
Alignment of nucleotide sequences of TR vs. VR1 and TR vs. VR2 for nd4-like (**A**) and nd12-like phages (**B**). Adenines of TR sequences susceptible to mutagenesis are highlighted in blue. Unusual substitutions arising from nucleotides other than A are highlighted in green. The amino acid sequence is shown above the TR sequence. Alignments were generated using ClustalW multiple alignment algorithm.

**Figure 10:**
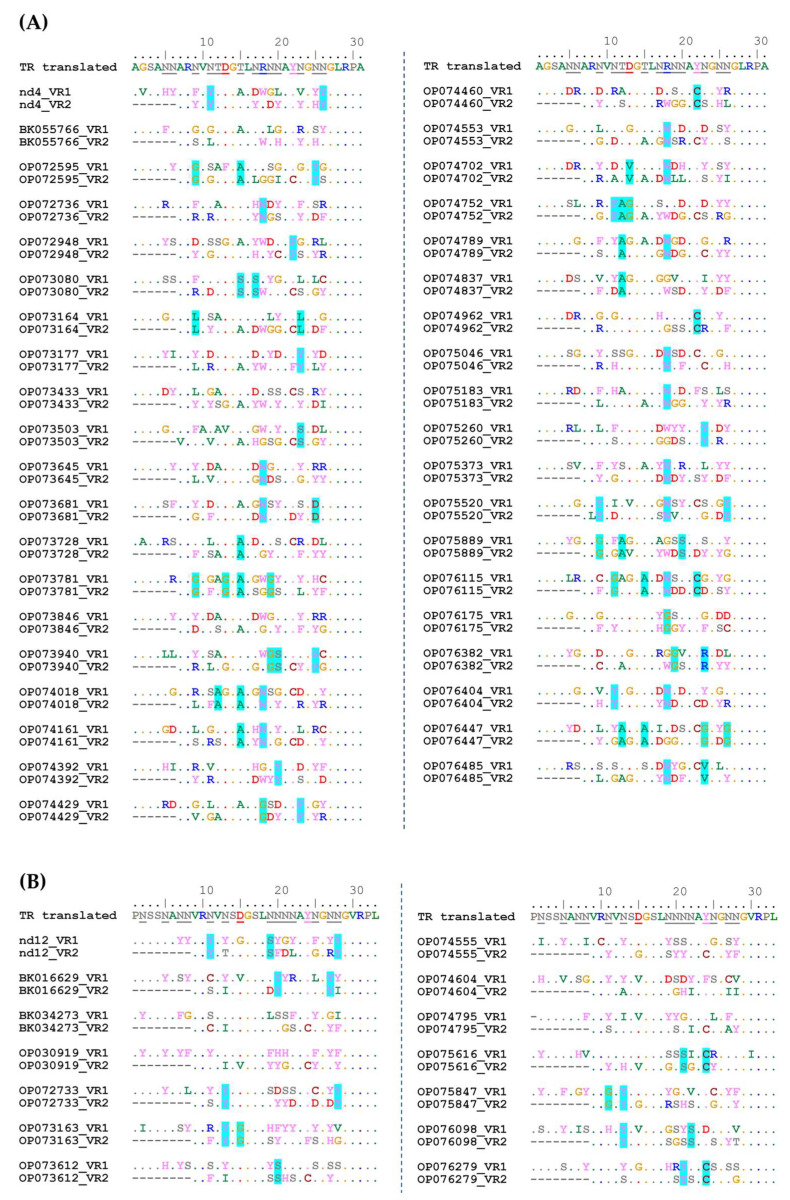
Alignment of aa sequences of TR, VR1, and VR2 in the genomes of the nd4-like (**A**) and nd12-like (**B**) phages. In TR, aa residues that can mutate as a result of retrohoming are underlined. Negatively charged aa are shown in red, positively charged aa in blue, aromatic aa in lilac, hydrophobic non-aromatic aa (except cysteine) in green, and hydrophilic uncharged aa in gray. The blue background marks the positions in which the same aa appeared as a result of mutagenesis and selection.

A total of 54 putative phage genomes with TR sequences identical to that in the nd4 genome were selected from the GenBank Nucleotide collection (nt) database. Analysis using VIRIDIC indicated that these phages probably belong to the same genus (intergenomic similarity > 87%). Only 39 of 54 genomes contained DGR cassettes with two target genes (Figure 9). TR in the nd4-like genomes contains 25 adenines (Figure 9). Of them, 24 positions were mutated in VR1 (in the target1 gene) in at least one of the nd4-like genomes (Figure 9). As a result, 13 aa were substituted in the tentaclin of nd4. In other nd4-like phages, from 7 aa to 19 aa substitutions in their tentaclins were identified (Figure 10). As for VRs of the target2 genes, all 21 adenines could be mutated, as VR2 is shorter. So, eight aa were substituted in the nd4_tgt2 protein and from 6 to 13 substitutions were found in the orthologous proteins of nd4-like phages (Figure 10).

As for the nd12-like genomes, 13 genome sequences were found that contained TR identical to that in the nd12 genome and two target genes in their DGR cassettes (Figure 9). A total of 28 adenines were found in TR in these genomes; all of them were mutated in VR1 and 20 adenines could be mutated in VR2. In the nd12 phage, 12 aa were substituted in the tentaclin (from 9 aa to 15 aa in the nd12-like phages) and 9 aa were mutated in the nd12_tgt2 protein (from 6 aa to 9 aa in other relative phages) (Figure 10).

In addition, along with substitutions A→N, there were 14 and 5 substitutions B→B (B = T, C, or G) found in both VR1 and VR2 sequences in the nd4-like and nd12-like genomes, respectively. Probably, these mutations appeared independently of retrohoming mediated by DGR.

It is noteworthy that in each examined phage genome, VR1 and VR2 sequences differed between themselves. Only sometimes, mutations coincided in corresponding positions in both VRs of the same phage genome.

Notably, most adenines are grouped in pairs in TRs of both nd4-like and nd12-like phages (Figure 9) and adenine in the second position of the AAC codons rarely changed to cytosine in contrast to adenine in the first position. Such an imbalance between the A→C mutations in the first and second positions can be caused both by a feature of hypermutagenesis for double adenines and the result of selection of preferred aa residues in target proteins. As for the AAT codons, which were found only in TRs of the nd12-like phages, both adenines infrequently changed to cytosine (Figure 9).

Since most adenines in the studied TRs are grouped into AAC and AAT codons (both encode Asn), 15 aa residues could appear as a result of hypermutagenesis (with the exception of *Gln*, *Met*, *Lys*, *Glu*, and *Trp*). However, substitutions for aromatic and charged (*Arg* and *Asp*) aa residues in the nd4-like phages and aromatic aa residues in the nd12-like phages are predominantly found. In addition, *Asn* is also often substituted by *Ser* in both groups of phages, unlike rare replacements for structurally similar *Thr* (Figure 10). Notably, there is a tendency to replace aa residue with *Cys* within the *TyrAsnGlyAsnAsn* motif of TR. According to 3D models of C-lec domains, this *Cys* appears close to another *Cys* residue outside the beta-hairpin. We suppose that such mutations lead to the formation of a disulfide bridge that stabilizes the C-lec domain (Figure 10 and Figure S7).

## 3. Discussion

In this study, we addressed the question of how one TR sequence can simultaneously be a template for two different VRs in the target genes found in the DGR cassettes of metagenomic phages nd4 and nd12. It is noteworthy that in both phages, the VR1 sequence is located in the 5′-terminal part of the target1 gene, whereas VR2 is found at the 3′-end of the target2 gene. In addition, no sequence similarity was observed between the target1 and target2 genes from each phage and 3D structure prediction indicated that the proteins encoded by the genes have different topology. However, both nd4_tgt1/nd12_tgt1 and nd4_tgt2/nd12_tgt2 proteins contain the C-lec domain, which is known to be involved in binding to certain proteins or oligosaccharides [27]. Importantly, the hypermutated site (VR) in the studied target genes encodes a beta-hairpin located on the surface of the C-lec domain (Figure 4, Figures S6 and S7). Probably, hypermutagenesis of VRs in both target proteins is required for these phages to adapt to the changing environment, including modification of the bacterial receptors profile.

Proteins encoded by the target1 genes of the nd4 and nd12 phages, in addition to the N-terminal C-lec domain, contain three Ig-like domains. Genes encoding proteins with a similar topology (N-terminal C-lec domain followed by Ig/Fn3-like domains) have been found in other MAGs (n > 350) and bacterial genomes (probably in prophages) and may contain from one to four Ig-like domains (Figure 6). In addition, similar genes have been previously identified in phage DGR cassettes [1,3,22]. However, due to the low quality of the generated 3D models, the C-lec fold for the N-terminal domain was not determined and specific VR-encoded aa residues on the domain surface were not localized. The use of more confident AlphaFold2-generated models in this study allowed us to reveal these details.

The presence of the C-lec domain with the hypermutated beta-hairpin indicates possible involvement of these proteins in receptor binding. A chain of several Ig-like domains gives overall flexibility to such molecules, similar to bacterial adhesins [25]. Given the prevalence of such proteins and their possible flexibility, we propose to call these proteins “tentaclins” (TENTACLe + proteIN). Importantly, the C-terminal anchor Ig-like domains of tentaclins have a clear consensus motif, despite the high diversity of the aa sequences of these domains (Figure 5A). Along with the specific topology, this motif can serve as a distinctive feature of tentaclins. As for the remaining Ig-like domains, it is not clear whether they are involved in additional binding to any molecules. So, a novel family of proteins with specific structure was discovered. These proteins contain the N-terminal C-lec domain with a specific beta-hairpin structure on its surface, followed by one to four Ig-like domains and the C-terminal Ig-like domain has a consensus motif. The tentaclin family is divergent and quite numerous and tentaclins occur in at least hundreds of phages.

Apparently, the topology of the tentaclins is favorable and has been repeatedly used during evolution. In addition, more complex proteins from phages and bacteria that have “tentaclin”-like organization were found. These molecules contain some additional elements aside from C-lec and Ig-like domains. Examples of such molecules are a protein from *Brevibacillus* sp. (GenBank id: NRS19645) containing an additional beta-propeller domain and a phage protein (BK023705) with the second C-lec domain at the C-terminus (Figure 6 and Figure S3). It should be noted that in the nd4-like and nd12-like MAGs containing only one target gene within the DGR cassette, this gene encoded the tentaclin in all cases. This fact confirms the importance of tentaclins.

As for the target2 genes of the nd4, nd12, and relative phages, they encode proteins that differ from tentaclins. Since nd4_tgt2 and nd12_tgt2 have a C-lec domain, they probably specifically recognize some bacterial structures. We hypothesize that the nd4_tgt2 and nd12_tgt2 proteins are involved in binding and infecting host cells, similar to the Mtd protein of the BPP-1 phage.

Comparison of the VR sequences between tentaclins and tgt2 proteins showed that these sequences differ both within the same phage and between related phages. The profile of selected mutations in both target proteins has a clear shift towards aromatic residues and *Ser* (for nd4-like and nd12-like phages) and also charged residues including *Asp* and *Arg* (for nd4-like phages). Apparently, this type of aa residues in hypermutated sites provides the best binding to yet unknown receptors recognized by these proteins.

It is unclear whether phages use their tentaclins and tgt2 proteins to bind to the host bacterium or if these proteins perform different functions. The first hypothesis is that only tgt2 proteins bind bacterial receptors, whereas tentaclins, like bacterial adhesins, are used for interaction with receptors on the surface of the intestinal epithelium, which allows phages to remain in the intestine. This hypothesis is supported by the fact that no similar pattern of mutated aa residues was found among VR sequences within the same phage. Moreover, some bacterial adhesins, such as invasin and intimin, have a similar organization—one C-lec domain and several Ig-like domains, although the C-lec domain is located at the C-terminus [28]. However, the profile of proteins and polysaccharides on the surface of intestinal cells is relatively constant, and hypermutagenesis of VR sequences in tentaclins is not required. The second hypothesis is that phages use tentaclins for interaction with immune cells that present in the intestine. These cells have a wide range of receptors and phages that interact with them can affect their immune response and thereby participate in the interaction between bacteria and macroorganism. In this case, hypermutagenesis of the VR sequences helps phages to adapt to the dynamic profile of immune cell receptors. The third hypothesis is that both tentaclins and tgt2 proteins bind to different receptors of one bacterial host or recognize different epitopes within the same receptor. Thus, it has been shown that the *Bordetella* BPP-1 and *Bordetella* BPP-6 phages recognize the same bacterial receptor pertactin, despite having different VR sequences in the Mtd protein [16].

In conclusion, the organization and role of two different target proteins from the same DGR cassette of metagenomic phages were investigated using bioinformatic methods. It was shown that one of the target proteins can be a member of a novel family of proteins—tentaclins. Tentaclins have a specific topology and the genes encoding tentaclins are relatively common in phage and bacterial genomes. The obtained data can be useful for further study of the mechanism of retrohoming and the molecular organization of phages that affect bacteria inhabiting the intestine.

## 4. Materials and Methods

### 4.1. Virome Sequencing

Viral DNA isolation from a fecal sample and DNA sequencing were performed as described previously [29]. Briefly, the sample from a healthy donor was resuspended in sterile phosphate-buffered saline and clarified by several consecutive centrifugations at 12,000× *g*. Five units of DNase I (Thermo Fisher Scientific, Waltham, MA, USA) were added to the final supernatant and the mixture was incubated for 4 h at 55 °C. Then, the mixture was treated with 100 μg/mL solution of Proteinase K (Thermo Fisher Scientific, Waltham, MA, USA) supplemented with 20 mM EDTA and 0.5% SDS for 3 h at 55 °C. DNA was purified using phenol–chloroform extraction with subsequent ethanol precipitation. The obtained DNA was diluted in 50 μL of 10% TE-buffer and, after measuring its concentration by Qubit 4.0 (Thermo Fisher Scientific, Waltham, MA, USA), applied for constructing a virome shot-gun library using the NEB Next Ultra DNA library prep kit (New England Biolabs, Ipswich, MA, USA). A MiSeq Benchtop Sequencer (Illumina Inc., San Diego, CA, USA) and a MiSeq Reagent Kit 2 × 250 v.2 (Illumina Inc., San Diego, CA, USA) were used for sequencing. The obtained sequences were assembled de novo using both the CLC Genomics Workbench software v.6.0 and SPAdes v. 3.15.

Sample collection was approved by the Local Ethics Committee of the Center for personalized medicine, Novosibirsk (protocol #2, 12.02.2019), where this sample was obtained. Written consent of the healthy volunteer was obtained according to guidelines of the Helsinki ethics committee.

### 4.2. Genome Analysis

All contigs longer than 10 kb obtained after assembly in SPAdes were used to search for similar sequences in the NCBI GenBank Nucleotide collection (nt) database using BLASTn. Sequences found to be similar to phage sequences were analyzed for the presence of portal protein and large terminase subunit genes. “Positive” sequences were analyzed for the presence of the DGR cassette using the myDGR online service (https://omics.informatics.indiana.edu/myDGR/ accessed on 17 August 2023) [20]. A search for nd4- and nd12-related sequences was performed using BLASTn and the NCBI GenBank nt database. Genome annotation was carried out using RAST server v. 2.0 (https://rast.nmpdr.org/ accessed on 23 September 2023) [30]. In addition, manual verification of the annotation results was carried out using the NCBI GenBank nr protein database. Comparative analysis of nd4 and nd12 genomic sequences was performed using the ViPTree version 3.7 web server (https://www.genome.jp/viptree accessed on 23 September 2023) with default parameters [31].

### 4.3. Analysis of Target1 and Target2 Gene Functions

BLASTp search, NCBI Conserved domain search (https://www.ncbi.nlm.nih.gov/Structure/cdd/wrpsb.cgi accessed on 1 October 2023), and HHPred search (https://toolkit.tuebingen.mpg.de/tools/hhpred accessed on 1 October 2023) were used to predict putative functions of proteins encoded by target1 and target2 genes. DALI server (http://ekhidna2.biocenter.helsinki.fi/dali/ accessed on 10 October 2023) was used to find structural similarity between AlphaFold2-generated models and experimental structures [32].

### 4.4. Modeling of Protein 3D Structure and Molecular Dynamics Simulation

3D models of proteins were predicted using ColabFold v. 1.5.3 implementation of AlphaFold2 program available at https://colab.research.google.com/github/sokrypton/ColabFold/blob/main/AlphaFold2.ipynb (accessed on 1 October 2023) [33]. Only models with a high degree of confidence (average pLDDT > 75) were used for the study. The models were visualized using UCSF Chimera, v. 1.13 [34].

Protein relaxation was performed using GROMACS v. 2020.3 [35] on Nvidia V100-equipped GPU nodes of the High Performance Computing Center of Novosibirsk State University (“NUSC NSU”). Molecular dynamics simulations were performed for 50 ns at 310 K and 1 bar pressure using the amber99SB force field and tip3p water molecules. Molecular dynamics trajectories were analyzed using VMD v. 1.9.3.

### 4.5. Analysis of Diversity of Tentaclin Genes

Sequences of C-terminal IgG domains (last 100 aa residues) of tentaclins of the phages nd4, nd12, FP_Mushu, FP_Brigit, and FP_Toutatis were used to perform PSI-BLAST search using NCBI Genbank non-redundant protein sequences (nr) database. The number of target sequences was chosen to be 1000, and the expectation threshold was 0.05. Non-redundant RefSeq proteins (records starting with “WP”) were excluded from results due to the inability to reference the original nucleotide sequence for such entries. Three consecutive iterations of the PSI-BLAST search were performed for each Cterm-IgG sequence. The results were downloaded as a single file; phage-related records were extracted using home-written python scripts. Then, all the records were combined, duplicates were removed, and corresponding phage nucleotide sequences were downloaded. Finally, a set of 383 phage sequences was divided into two parts (due to limitations of online version of VIRIDIC), which were used for intergenomic similarity calculation using VIRIDIC (https://rhea.icbm.uni-oldenburg.de/viridic/ accessed on 1 October 2023) [36]. Finally, these two parts were reorganized so that the largest groups were in the first heatmap, whereas the smaller groups and individual sequences were in the second heatmap.

### 4.6. Analysis of Diversity of VR Sequences

BioEdit 7.2.5 [37] and AlignX (a tool from Vector NTI suite 8.0) were used for performing nucleotide and amino acid sequence alignment, as well as for calculation of the sequence identity.

## Data Availability

Raw NGS data containing nd4 and nd12 sequences are available at Genbank (Bioproject PRJNA1027629). The nd4 and nd12 sequences were deposited to GenBank, accession numbers: OR777945 and OR777946.

## Supplementary Materials

The following supporting information can be downloaded at: https://www.mdpi.com/article/10.3390/ijms242417324/s1.
