# Supplementary material for: Tentaclins—A Novel Family of Phage Receptor-Binding Proteins That Can Be Hypermutated by DGR Systems
Source: Int J Mol Sci. 2023 Dec 10;24(24):17324. doi: 10.3390/ijms242417324 (PMC10743442; doi:10.3390/ijms242417324)
Supplement: Supplementary file 1 [file ijms-24-17324-s001.zip › Supplementary figures.pdf]

Supplementary information

Figure S1: AlphaFold2-generated models of nd4_tgt1, nd4_tgt2, nd12_tgt2, nd12_tgt2, and related proteins colored based on pLDDT value, indicating high overall confidence of the models.

Figure S2: VIRIDIC heatmap indicating intergenomic similarity between tentaclin gene-containing phage-like sequences from second part of dataset (178 sequences of 373).

Figure S3: AlphaFold2 model of unusual 1180aa-long tentaclin-related protein from *Brevibacillus* sp., protein accession number NRS19465.

Figure S4: Schematic view of DGR cassettes for phage sequences randomly selected from the five largest groups obtained using VIRIDIC analysis.

Figure S5: Structural and amino acid alignments of VR regions of proteins nd4_tgt1, nd4_tgt2, nd12_tgt1, and nd12_tgt2.

Figure S6: Surface representation of C-lec domains of nd4_tgt1 and nd4_tgt2 proteins and nd4_tgt1 tentaclin molecule.

Figure S7: Models of the C-lec domains of the proteins nd4_tgt1, nd4_tgt2 and nd12_tgt1, showing the close location of cysteine residues in the beta-hairpin to cysteine residues from the lectin core.

Data S1: Annotation of nd4 genome.

Data S2: Annotation of the nd12 genome.

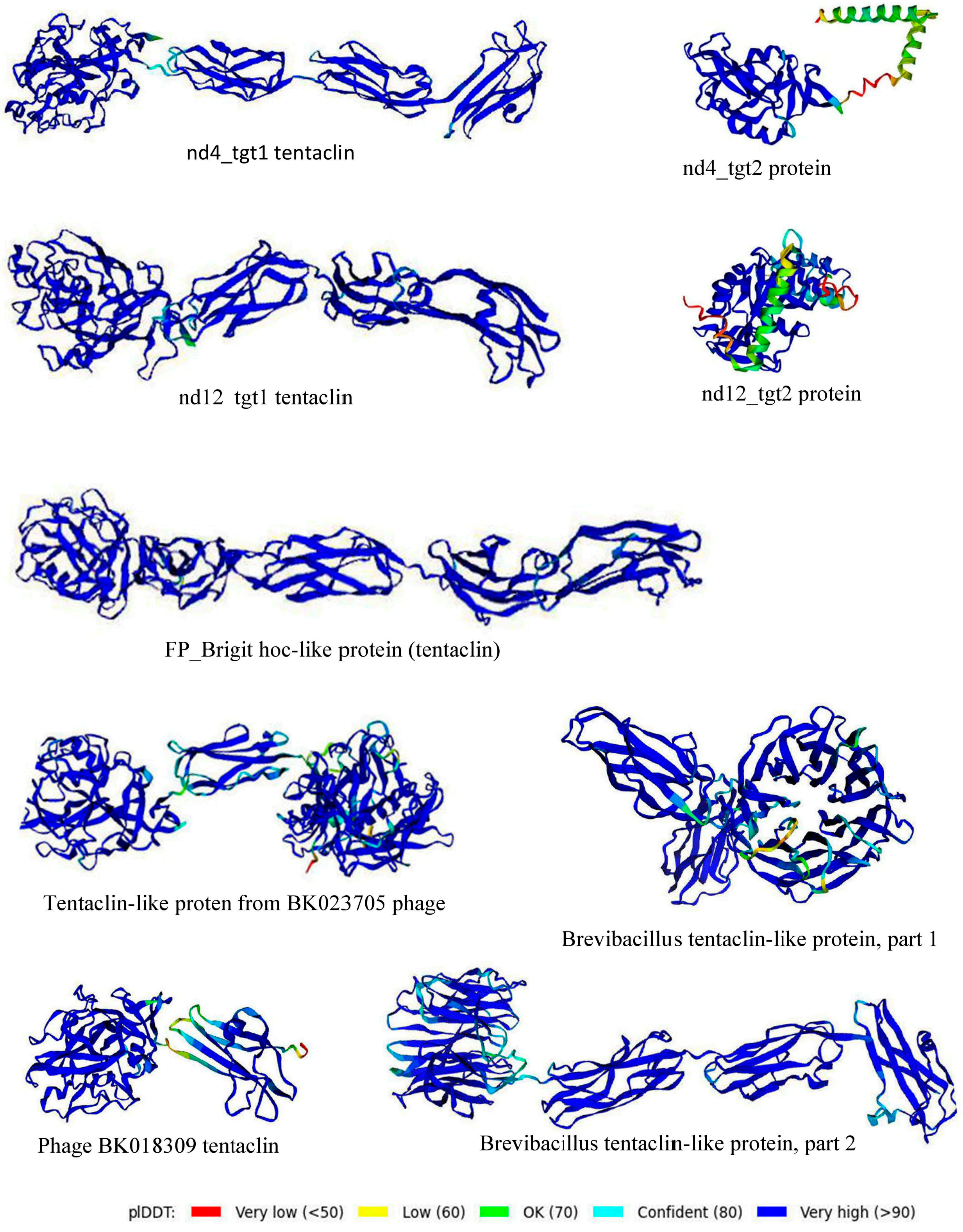

Figure S1. Alphafold2-generated models of nd4_tgt1, nd4_tgt2, nd12_tgt2, nd12_tgt2 and related proteins colored based on pLDDT value, indicating high overall confidence of the models.

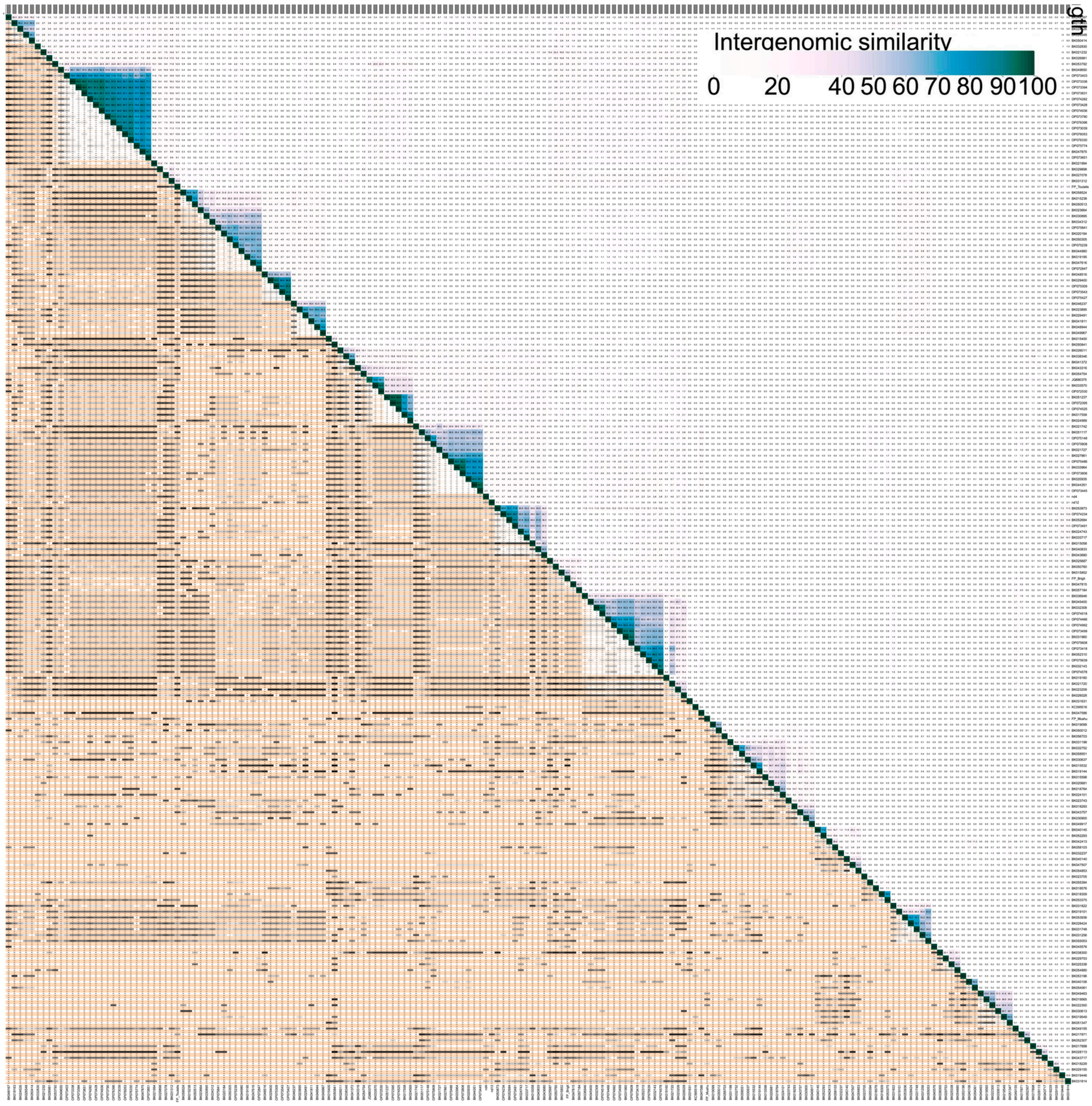

Figure S2. VIRIDIC heatmap indicating intergenomic similarity between tentaclin gene-containing phage-like sequences from second part of dataset (178 sequences of 373).

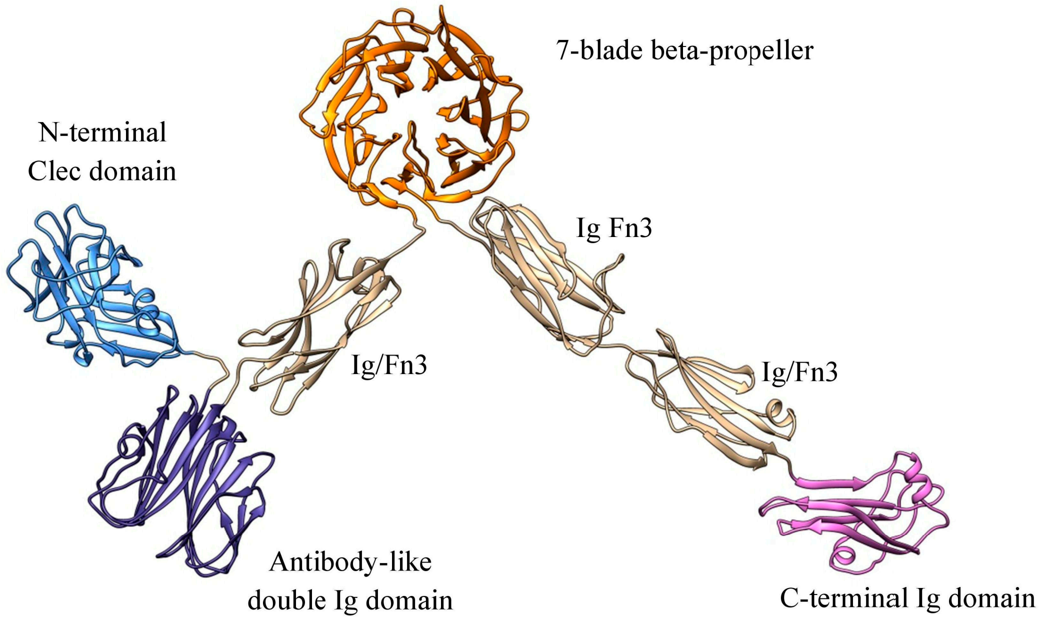

Figure S3. Alphafold2 model of unusual 1180aa-long tentaclin-related protein from *Brevibacillus* sp., protein accession number NRS19465. C-terminal Ig domain (shown in pink) have consensus sequence characteristic for tentaclin Cterm-Ig domains.

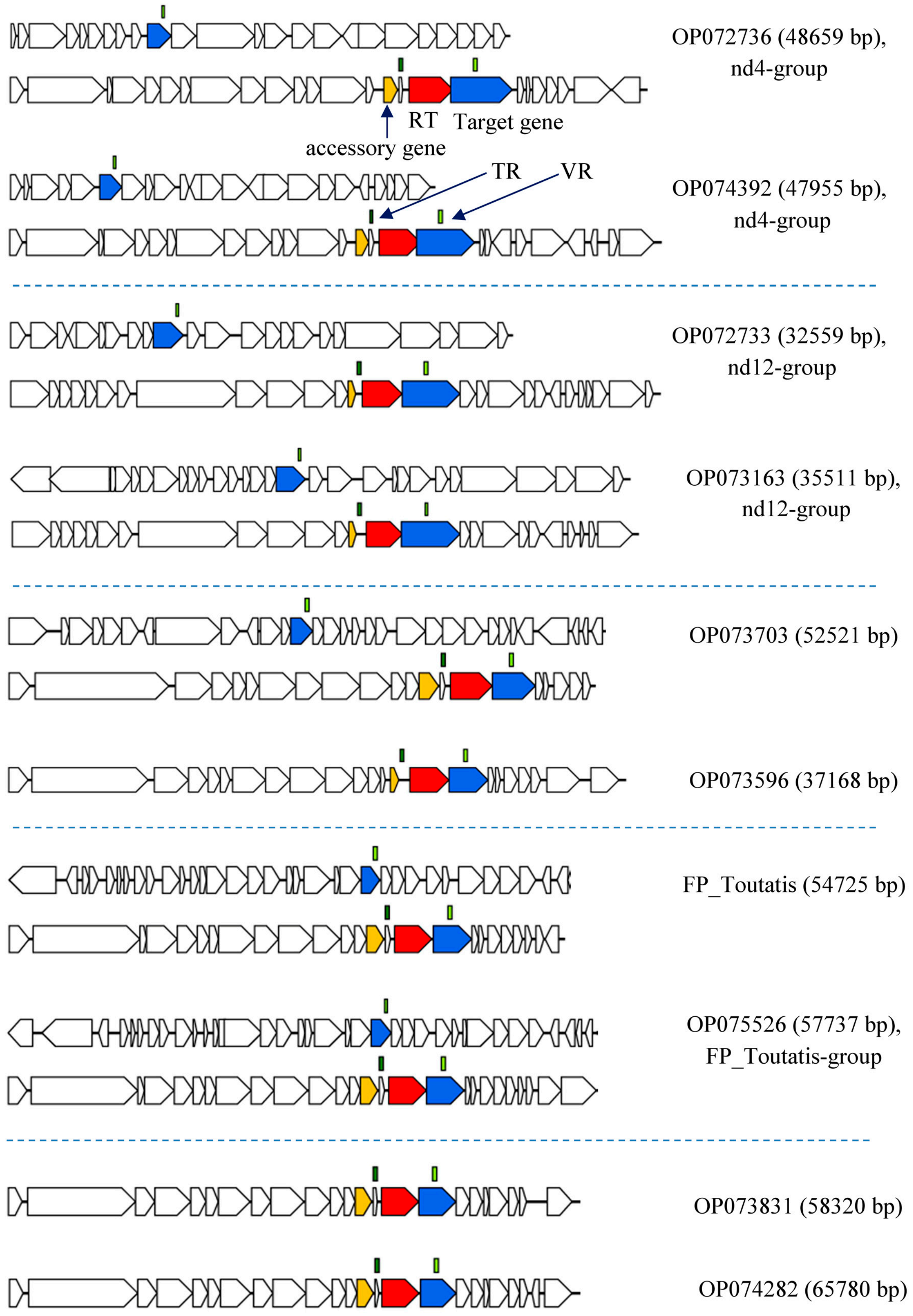

Figure S4. Schematic view of DGR cassettes for phage sequences randomly selected from the five largest groups (separated by dashed lines) obtained using VIRIDIC analysis (Figures 7 and S2). Target genes are colored in blue, reverse transcriptase (RT) genes are red, accessory protein genes are orange. Template repeats (TR) are shown as small dark-green boxes, variable repeats (VR) are shown as small light-green boxes. Sequence names are listed on the right.

(A)

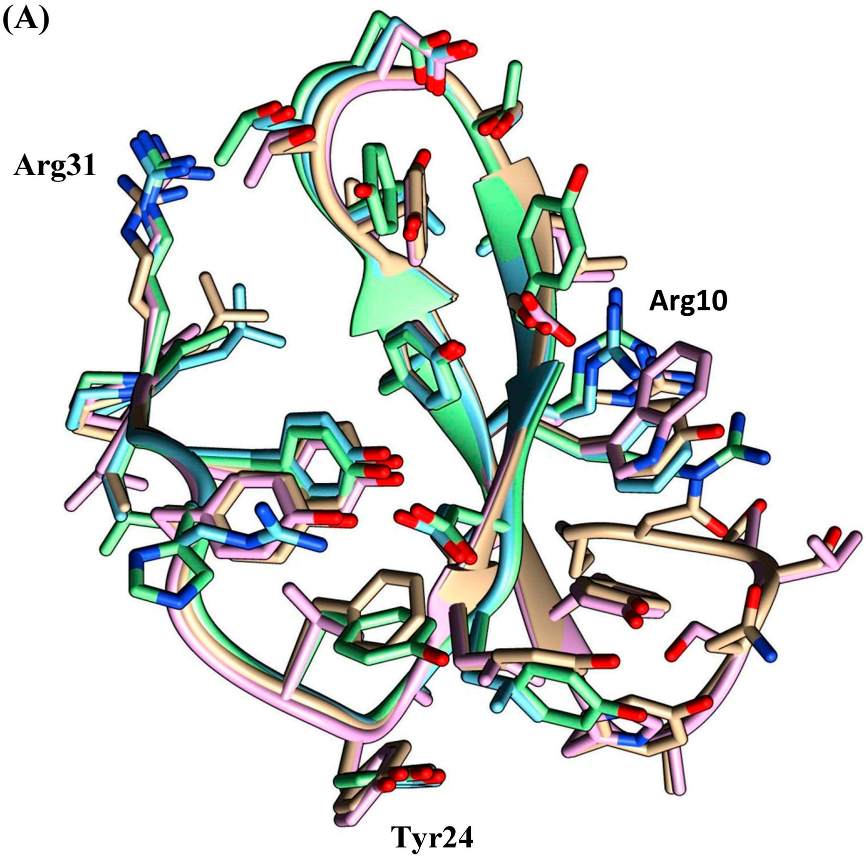

(B)

```
                                  10        20        30
                         ....|....|....|....|....|....|...
nd4_tgt1_VR    --AVSAHYARFVYTDGALDWGLAYVGYYGLRPA
nd4_tgt2_VR    --------ARYVYTDGTLYRDYAYYGHYGLRPA
nd12_tgt1_VR   PNSSNAYYVRYVYSGGSLSYGYAYFGYYGVRPL
nd12_tgt2_VR   --------VRYVTSDGSLSFDLAYGGRYGVRPL
```

Figure S5. Structural (A) and amino acid alignments (B) of VR regions of proteins nd4_tgt1, nd4_tgt2, nd12_tgt1 and nd12_tgt2. Labeled residues are numbered according to (B).

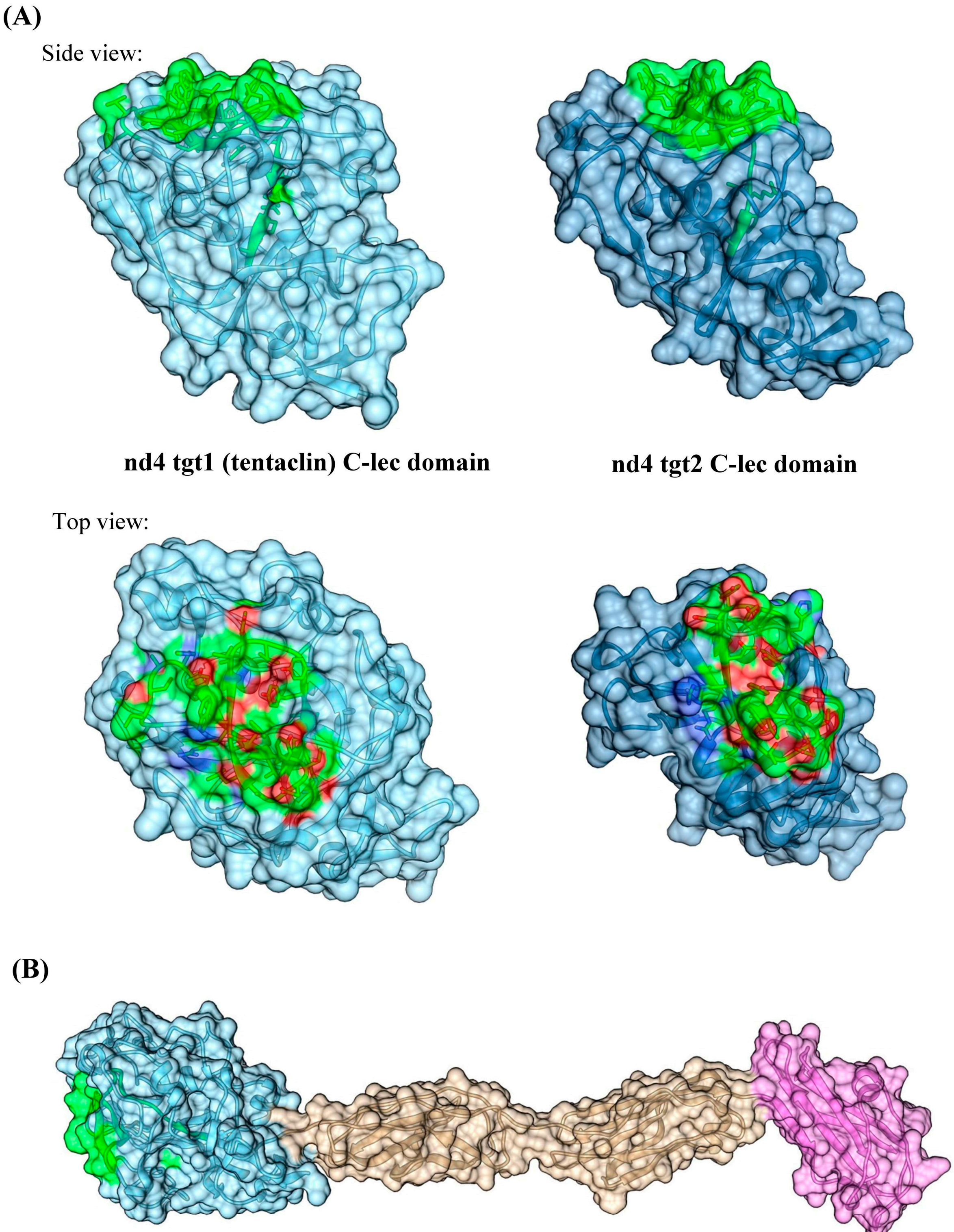

Figure S6. Surface representation of C-lec domains of nd4_tgt1 and nd4_tgt2 proteins (A) and nd4_tgt1 tentaclin molecule (B). Models under subheading A are shown to the same scale. C-lec domains are shown in blue, C-terminal Ig domain is in pink, other Ig domains are in tan. Beta-hairpins encoded by VR regions are in green. Heteroatom coloring was also applied to the models shown in the top view: oxygens are red, nitrogens are blue.

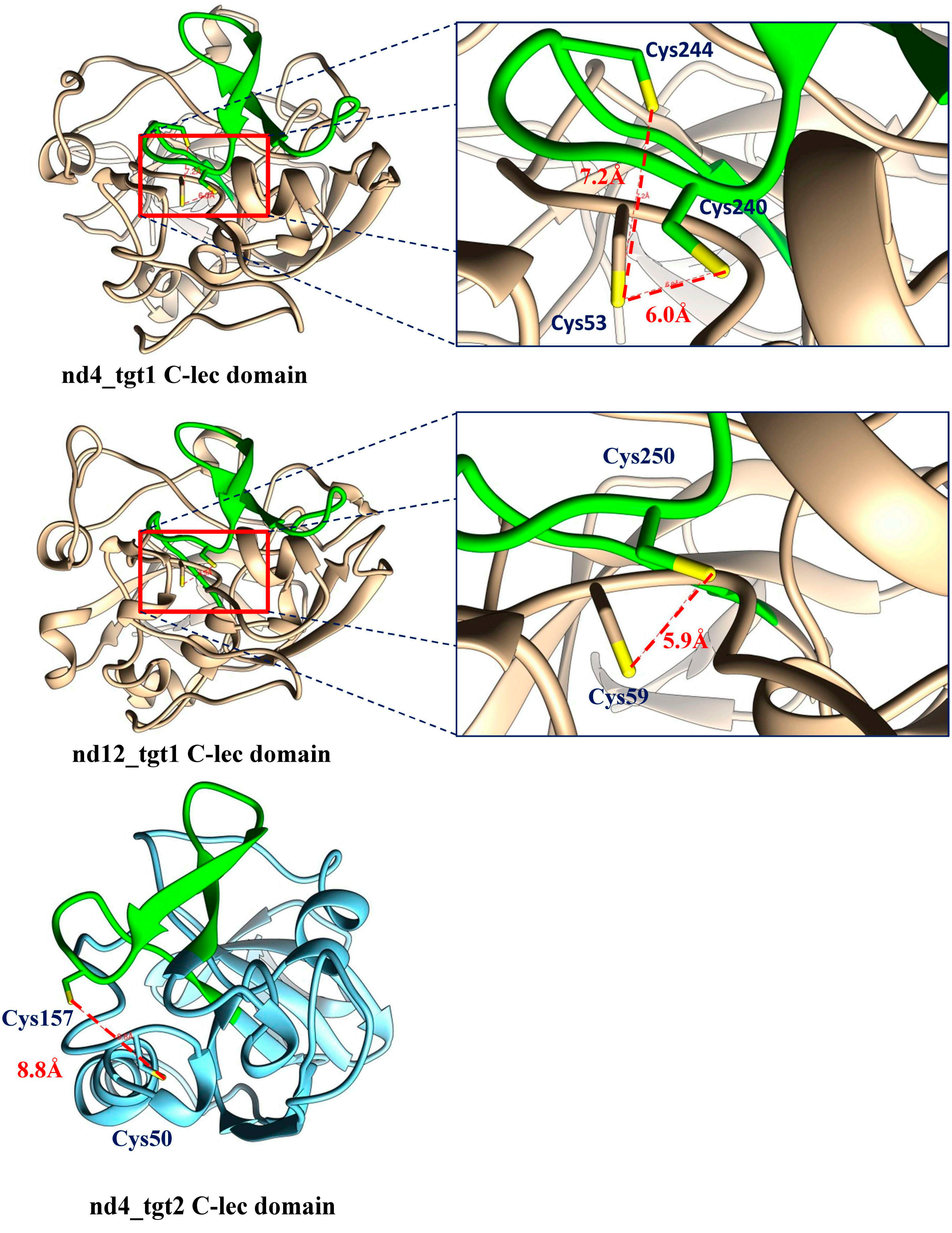

Figure S7. Models of the C-lec domains of the proteins nd4_tgt1, nd4_tgt2 and nd12_tgt1, showing the close location of cysteine residues in the beta hairpin (shown in green) to cysteine residues from the lectin core. Sulfur atoms are shown as yellow sticks.
